# Psychometric Properties of the Positive Thinking Skills Scale (PTSS) among Portuguese Adults

**DOI:** 10.3390/bs13050357

**Published:** 2023-04-25

**Authors:** Telma Catarina Almeida, Ionela Catalina Ifrim

**Affiliations:** 1 Egas Moniz School of Health and Science, Instituto Universitário Egas Moniz (IUEM), 2829-511 Caparica, Portugal; 2 CiiEM—Centro de Investigação Interdisciplinar Egas Moniz, Instituto Universitário Egas Moniz (IUEM), 2829-511 Caparica, Portugal; 3 LabPSI—Laboratório de Psicologia Egas Moniz, Instituto Universitário Egas Moniz (IUEM), 2829-511 Caparica, Portugal

**Keywords:** positive thinking, psychometric properties, resilience, ruminative negative thinking

## Abstract

Background: Positive thinking is a cognitive attitude that focuses on optimism and aims for positive results. Positive thinking leads to positive emotions, more adaptive behaviors, and better problem solving. Positive thoughts can inspire individuals and have been linked to increased psychological health. On the other hand, negative thoughts are related to unsatisfactory mental health. Objectives: This study aimed to analyze the factor structure and psychometric properties of the Portuguese version of the Positive Thinking Skills Scale (PTSS) and to verify the correlations between positive thinking, resilience, and repetitive negative thinking. Participants: The sample comprised 220 Portuguese participants between 18 and 62 years of age (*M* = 24.9, *SD* = 6.58), and the majority were women (80.5%). Method: Participants responded to an online sociodemographic questionnaire, the PTSS, the Persistent and Intrusive Negative Thoughts Scale (PINTS), and the Resilience Scale-10 (RS-10). Results: Confirmatory factor analysis results indicated that the original one-factor structure of the PTSS obtained good fits. An excellent value of internal consistency was found. The results also revealed convergent and discriminant validity. Conclusion: The PTSS is a brief and reliable instrument for assessing positive thinking skills, and its use in research is recommended.

## 1. Introduction

Positive thinking is a cognitive process that focuses on the optimistic side of reality [1], creating hopeful images [2]. Positive thinking does not ignore realistic appraisals [1,2] but focuses on the positive aspects of events, positively influencing emotions and behaviors and increasing happiness. Focusing on positive information can lead to a positive mood and more recurring favorable judgments [3], as well as one’s belief in the possibility of overcoming difficulties or obstacles [1]. Positive thinking enables a person to envision a brighter future [2]. It is desirable to have good things, to anticipate positive consequences, and to have hope for a promising future [4]. According to McGrath [5], positive thinking is a vast term that refers to an attitude that manifests in one’s thoughts, actions, feelings, and words. A positive mental attitude allows for one’s thoughts to promote growth, expansion, and fulfillment. Positive thinking has various effects, including positive feelings, emotions, better behavioral qualities, and problem-solving skills [2,6].

Most previous studies have focused on the pathological functioning of individuals, ignoring positive aspects and their potential to promote well-being [7]. Nevertheless, some perspectives, such as that from Positive Psychology, emphasize optimism and positive thinking, focusing on human problems, such as individuality, being, self-actualization, becoming, hope, health, love, creativity, and meaning [8]. Positive thinking potentiates physical and mental health, effective coping behaviors, positive humor, and positive responses to psychological interventions [9]. Positive interventions in mental health improve one’s resources to develop resilience [10]. Some researchers have examined the psychological and physical benefits of positive thinking, positive feelings, positive emotions, and positive behavioral traits [11,12]. One of the essential functions of positive emotions (influenced by positive thinking) [3] is in helping the individual to prepare for future challenges [11]. Lyubomirsky and King [13] argue that people who experience positive emotions tend to create and achieve new goals in life. People who approach situations with optimism assess stressful situations as manageable and use efficient coping strategies to help solve problems. Positive thinkers believe that their lives are going well and that their resources are adequate, since they perceive that their goals in life are being met [14].

Positive thinking and stress have an inverse relationship [15]. The presence of daily positive thinking leads to positive emotions that help to reduce stress reactivity. Positive thinking can attenuate the adverse consequences of stress. Individuals who use positive thinking show less vulnerability to stressful circumstances, allowing them to cope better than those with negative thoughts [6]. Stress has been linked to heart disease [16], infectious illness [17], and autoimmune disorders [18]. Positive thinking and positive emotions are related to decreasing distress and increasing health [19]. In stressful circumstances, there are two significant advantages to thinking positively. The first is that positive thinking will help individuals to cope better. The second is that thinking positively enhances the chances of a favorable outcome. Optimism has been positively linked to higher levels of self-reported vitality and mental health and lower levels of depression [20], and positive thinking decreases anxiety levels [21].

Cognitive behavioral therapy focuses on conscious cognitive reappraisal as the primordial change mechanism [22]. One of the goals of cognitive behavioral therapy is to access cognitive appraisals to detect negative thoughts that may underlie the difficulty in emotion regulation and maladaptive behaviors [1,23], educating patients about the impact of negative thoughts and negative self-talk. According to the cognitive behavioral intervention model, identifying cognitive appraisals and implementing positive thought-training work is essential to establishing greater effectiveness, for example, in treating anxiety and chronic mental illness and building self-confidence. Individuals usually assume that their thoughts are accurate. For example, depressed individuals tend to see only the facts that support their negative view (e.g., their mistakes) and to ignore the positive things about themselves or that which they possess. For this reason, it is important to detect negative thoughts and to identify the facts that support a more positive view (e.g., one’s qualities) [23]. One way to utilize the techniques that allow an individual to use cognitive reappraisal in problems connected to anxiety or depression is to look for the opportunity instead of the negative argument in a situation, focusing on positive perspectives of reality [1].

Resilient individuals experience less depression and report increased psychological growth [11]. Resilient individuals are emotionally positive, approach life with vigor and energy, and are eager and open to new experiences [6]. They cope by using positive thinking and humor [24,25]. In addition, resilient people nurture happy sentiments in themselves and inspire positivity in those around them, building a supportive social system that aids the use of adaptive coping strategies [26]. Positive thinking directly impacts college students’ suicide resilience [27]. Resilience is associated with beneficial developmental adjustments in the face of adversity. As a result, identifying resilience processes requires definitions of adversity and positive adaptation [28].

### 1.1. Positive and Negative Thinking

Positive thinking has been related to increased mental health, but negative thinking can decrease psychological health. Positive thoughts can inspire individuals, whereas negative thoughts are linked to unsatisfactory health outcomes [6]. Positive thinking is learned through life and can be strengthened over time [29]. Negative thinking is positively correlated with psychological maladjustment (e.g., anxiety, depression, stress, and anger) and is negatively correlated with psychological well-being (e.g., happiness and life satisfaction). However, positive thinking positively correlates with happiness, life satisfaction, and psychological well-being and negatively correlates with psychological maladjustment [30].

Repetitive negative thinking is linked to emotional distress based on past, present, and/or future concerns and previous and/or predicted unpleasant experiences. Additionally, this thinking is recurrent, often obtrusive, challenging to resolve, and leads to dysfunctional outcomes [31]. Previous repetitive negative thinking research has focused on disorder-specific cognitive constructs, such as rumination in depressive disorders and worries in anxiety disorders [32,33]. Although differences have been described between rumination and worry, in which rumination is related to negative thoughts and worry is linked to thoughts about possible adverse consequences, the constructs are correlated and comorbid [32]. Comorbidity in mental diseases is common, harms people’s well-being and quality of life, and considerably reduces their functional areas [34].

### 1.2. The Positive Thinking Skills Scale

The Positive Thinking Skills Scale (PTSS) was developed by Bekhet and Zauszniewski [2] with a sample of 109 caregivers of individuals with autism in the USA. According to the authors, the construction of the PTSS was based on cognitive behavioral theory, revising the existing positive thinking instruments and using a literature review (see [2] for more details). The PTSS includes items that focus on transforming negative into positive thinking and supporting positive thoughts [2].

The results showed that the PTSS has a one-factor structure with eight items and was internally consistent (α = 0.90). The PTSS is a reliable and valid scale that measures the frequency of use of positive thinking skills. The scale has demonstrated significant correlations with positive cognitions, resourcefulness, depression, and general well-being, meaning it has construct validity. A higher score suggests that positive thinking skills are engaged more frequently, whereas a lower number suggests they are engaged less frequently [2]. In 2019, Matel-Anderson and Bekhet studied the psychometric properties of the PTSS in a sample of 131 undergraduate college students in the USA [27].

In 2015, the PTSS was translated into Turkish in a study involving 295 college students. The scale proved to be valid and reliable, with an internal consistency coefficient of 0.87 [35]. The PTSS was also translated into Arabic in 2020 and used in a sample of 100 Middle Easterner first-generation immigrants who were 18 years of age or older. The instrument also proved to be valid and reliable in Arabic research, with an internal consistency coefficient of 0.89 [36].

The PTSS has demonstrated positive correlations with other constructs, such as resilience [37], perceived social support, self-esteem [27], positive emotions, resourcefulness, and general well-being, as well as negative correlations with depression [2] and suicide resilience [27]. Furthermore, some studies show that positive thinking training reduces mental health problems [38,39].

In Portugal, there is no other instrument that evaluates this construct, and the literature shows the importance of evaluating and training positive thinking skills to develop resilience [37], efficient problem-solving [2], and well-being [7]. Therefore, this study aimed to analyze the factor structure and psychometric properties of the Portuguese version of the PTSS and to verify the correlations between positive thinking, resilience, and persistent and intrusive negative thoughts.

## 2. Materials and Methods

### 2.1. Participants

The sample comprised 220 Portuguese participants (Table 1) between 18 and 62 years of age (*M* = 24.9, *SD* = 6.58), and the majority were women (*n* = 177, 80.5%). Most of the participants of this sample had a higher education (*n* = 139, 63.2%), and the remainder finished high school (*n* = 76, 34.5%) and elementary school (*n* = 5, 2.3%). From the total sample, 106 (48.2%) participants were still students, 95 (43.2%) were employed, and 19 (8.6%) were unemployed.

Regarding sexual orientation, 178 (80.9%) described themselves as heterosexual, 23 (10.5%) as bisexual, 7 (3.2%) as homosexual, 5 (2.3%) as pansexual, and 7 (3.2%) were undefined. From the total sample, 85.5% (*n* = 188) participants were single, 12.7% (*n* = 28) were married, and only 1.8% (*n* = 4) had gone through a divorce.

### 2.2. Measures

Sociodemographic Questionnaire (SQ). The Sociodemographic Questionnaire covers questions about sex, age, nationality, education, sexual orientation, marital status, and professional situation.

Positive Thinking Skills Scale (PTSS) [2]. The PTSS is a self-response scale that measures how often people use their positive thinking skills. This instrument consists of 8 items, rated on a 4-point Likert scale (0—never, 1—sometimes, 2—most of the time, 3—always). The final score can range from 0 to 24 points, with a higher score indicating that positive thinking skills are used more frequently and a lower score indicating the opposite. Higher scores on the PTSS represent more positive thinking skills, increasing positive thoughts and eliminating or modifying negative ones. On the other hand, low scores indicate less positive thinking skills. Regarding internal consistency, the results revealed a high Cronbach’s alpha coefficient (α = 0.90), thus showing high reliability. In the present sample, the reliability levels were good.

Persistent and Intrusive Negative Thoughts Scale (PINTS) [40]. The Portuguese version of the PINTS [41] was used. This self-report instrument aims to assess a thinking style characterized by persistent thoughts that are also intrusive and negative. It consists of 5 items rated on a 5-point Likert scale (1—never, 2—almost never, 3—sometimes, 4—often, 5—almost always). The score ranges from 5 to 25. Higher scores on the PINTS indicate more repetitive negative thinking. The results revealed a very satisfactory Cronbach’s alpha coefficient (α = 0.88) regarding internal consistency. In the present sample, the reliability levels were good (α = 0.90).

Resilience Scale-10 (RS-10) [42]. The RS-10 is a self-response instrument that aims to assess the level of resilience in the adult population. It consists of 10 items rated on a 5-point Likert scale (1—never, 2—rarely, 3—sometimes, 4—almost always, 5—always). The score ranges from 10 to 50. High scores indicate high levels of resilience. Regarding internal consistency, the results revealed a satisfactory Cronbach’s alpha coefficient (RS-10 total α = 0.86, self-determination α = 0.84, adaptability α = 0.81). The present sample’s reliability levels were good (RS-10 total α = 0.91, self-determination α = 0.86, adaptability α = 0.85).

### 2.3. Procedure

The study design was cross-sectional and used a non-probabilistic sample. First, we translated the PTSS from English to Portuguese. The translation was performed by three researchers who are fluent in both languages. After that, the Portuguese translation was retranslated into English by two different researchers. Two researchers who are fluent in both languages rectified the differences by agreeing to eliminate semantic differences between versions of the PTSS (i.e., English and Portuguese). We tested the final version on 25 Portuguese participants to guarantee they understood the PTSS version used in this study. We did not make additional changes to the PTSS, since the results from the pilot analysis were satisfactory (i.e., none of the items presented questions to the participants; Cronbach’s alphas of 0.93 for the PTSS). The final versions of the PTSS and the other instruments were inserted in a Google Form. We distributed the online link to fill the research protocol via social networks (e.g., LinkedIn and Facebook) and e-mail (e.g., researchers’ contacts and university and institutional mailing lists), so that we could acquire a sample that included as many participants as possible from several regions of the country. We invited individuals with Portuguese nationality over 18 years to participate in the study. The eligibility criteria for participants were: (1) knowing how to read and write, (2) age over 18 years old, and (3) Portuguese nationality. All participants signed an electronic informed consent form before concluding the research protocol through the web-based survey.

All the instruments were applied in the Portuguese language. Participation in the study took approximately 10 min and was voluntary and anonymous (i.e., no personal information was collected). The participants did not receive financial support, compensation, or incentives to participate in the study. All the ethical principles outlined in the Declaration of Helsinki [43] were followed. The study was approved by the Ethics Committee of the Egas Moniz School of Health and Science.

### 2.4. Data Analysis

We used descriptive statistics to characterize the sample. Since there was a prior hypothesis of the structure of the scale, Confirmatory Factor Analyses (CFA) using the software IBM SPSS Amos (version 28.0.0) were conducted to test the factor model in our sample. We used the maximum likelihood and selected the maximization history, standardized estimates, squared multiple correlations, factor scores weights, tests for normality and outliers, covariances of estimates, correlations of estimates, and modification indices. To assess the adjustment quality, the following indexes were used: χ^2^/df (ratio of chi-square on degrees of freedom) inferior to 2.0; GFI (Goodness of Fit) superior to 0.90; CFI (Comparative Fit Index) superior to 0.90; and RMSEA (Root Mean Square Error of Approximation) inferior to 0.10 [44].

The Statistical Package for Social Sciences (SPSS; IBM SPSS Statistics, version 28.0, Armonk, NY, USA: IBM Corp.) was used to perform other statistical analyses. Correlations with other constructs were assessed by testing the Pearson correlation coefficient between PTSS scores, the PINTS, and the RS-10. Construct validity was assessed through the Extracted Average Variance (VME; ≥0.5) [45] and Composite Reliability (CR; ≥0.7) [42]. The internal consistency was calculated to analyze the instrument’s psychometric properties through Cronbach’s alpha (≥0.7) [46].

## 3. Results

### 3.1. Preliminary Analyses

The PTSS did not contain missing values or outliers. There was no evidence of kurtosis (−0.414 to −0.781) or skewness (−0.037 to 0.142), because all values fell within the acceptable range of ±2 [47]. Because the data were normally distributed, all the analyses were conducted using parametric statistics.

### 3.2. Confirmatory Factor Analysis

**Figure 1:**
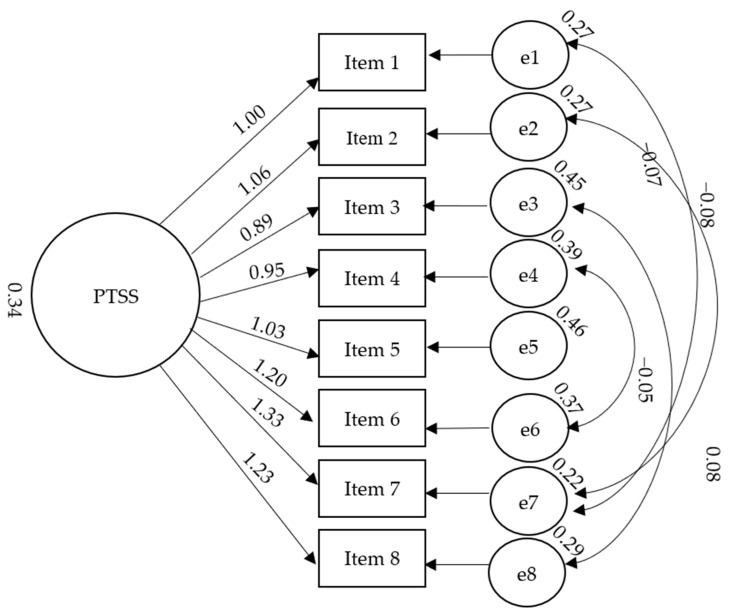
Correlated one-factor structure of the PTSS.

We tested the PTSS one-factor structure (Figure 1) through confirmatory factor analysis (CFA). The analysis of the fit indices revealed that the model was adequate (χ^2^(16) = 54.72: CFI = 0.957; NFI = 0.941; RMSEA = 0.100; GFI = 0.941). All the items’ factor loadings were higher than 0.50 (Table 2).

### 3.3. Reliability

A Cronbach’s alpha value of 0.90 indicated that the eight-item PTSS had satisfactory to excellent internal consistency. All the PTSS items were well correlated (Table 3), and the item-total coefficients suggested that deleting any of the items would not significantly improve the scale’s reliability.

### 3.4. Convergent and Criterion Validity

To assess the convergent analysis of the PTSS, Pearson’s coefficient correlations between the PTSS and resilience were performed. To assess the divergent analysis of the PTSS, the Pearson’s coefficient correlations between the PTSS and persistent and intrusive negative thoughts were calculated.

As shown in Table 4, the PTSS was significantly and positively correlated with the RS-10 (self-determination and adaptability) and negatively correlated with the PINTS.

To better explore the construct validity, the Average Variance Extracted (AVE) and the Composite Reliability (CR) of each factor were estimated. The AVE was satisfactory (0.53; ≥0.5 cf. Netemeyer et al., 2003), and the CR was above the minimum recommended (0.92; ≥0.7; cf. Netemeyer et al., 2003).

## 4. Discussion

This study aimed to analyze the factor structure and the psychometric properties of the Portuguese version of the PTSS and to verify the correlations between positive thinking, resilience, and persistent and intrusive negative thoughts. Since there are no validated positive thinking skills measures for the Portuguese population, and PTSS has been related to resilience and well-being [7,37], an adaptation of the PTSS for this population is of extreme importance.

The PTSS was developed firstly to measure the frequency of using positive thinking skills [2] and to assess which positive thinking skills are used by college students [27]. In this study, the confirmatory factor analysis replicated the one-factor model proposed by Bekhet and Zauszniewski [2], confirming the results from the original version of the PTSS. Our results support using the PTSS to measure positive thinking skills [2,27]. In terms of reliability, the results of this study replicated the findings of the original one, showing that the PTSS has good internal consistency with a high Cronbach’s alpha. Previous research reported that the PTSS presented a good Cronbach’s alpha in a sample of 109 caregivers of individuals with autism [2]. In another study using the PTSS in Turkish [35], the authors also reported a good Cronbach’s alpha in a sample of 295 Turkish university students. Lastly, the PTSS was used in the Arabic 100 Middle Easterner first-generation immigrants’ sample, also showing a good alpha [36]. In this study, the goodness-of-fit indices showed good scores, except for the RMSEA, which scored an acceptable measure.

This research indicated that the factor loadings of the scale in Portuguese adults ranged from 0.69 to 0.85, confirming that the PTSS is a valid measure, meaning that it measures the construct it is supposed to measure. The construct validity of the PTSS was supported in our study of 220 Portuguese adults. This result also confirmed the data from the original version of the PTSS [2].

Convergent and divergent validity results showed that the PTSS was significantly positively correlated with resilience and significantly negatively correlated with ruminative negative thinking, meaning that the construct validity is fit. In the research conducted by Matel-Anderson and Bekhet [27], the PTSS also correlated positively with the construct of resilience. The authors argue that positive thinking is a way of helping to build resilience [27]. On the other hand, positive thinking has been negatively correlated with mental health problems [38]. We assessed the reliability of the PTSS by examining inter-item correlations, in which every correlation was statistically significant and positive. This finding corroborates the original psychometric study of the PTSS [2].

Some studies have found that positive thinking can help to increase mental health, whereas negative thinking can decrease mental health [5]. Positive thoughts motivate individuals, whereas negative thoughts lead to the opposite outcome [6]. Persistent and intrusive negative thoughts lead to more problems. On the other hand, people who use positive thinking can better overcome their problems. In this sense, studies have shown the importance of individual thought patterns in developing healthy life satisfaction [48].

Positive approaches are very important in Psychology. Positive psychology focuses on skills that lead to pleasure, optimism, and problem solving instead of emphasizing weaknesses [49]. Positive approaches identify training methods and processes, with positive thinking leading to individual well-being, happiness, and better life satisfaction. Using techniques that focus on positive thinking can increase resilience, and using a positive strategy can be a barrier to emotional and physical harm [50]. Using positive thinking instead of persistent and intrusive negative thoughts significantly increases resilience and psychological skills [51]. Resilient people report increased psychological growth and experience less depression [11]. They are distinguished by positive emotionality and are reportedly more open and eager to new experiences [6].

This study has some limitations that should be mentioned. First, as participants responded to the study online, only individuals with internet access could respond. Furthermore, in online data collection, we cannot control the environment in which the participant responds to the study (e.g., light, background noise, whether they are accompanied, or whether they have privacy). Additionally, since validity in online research is a well-studied concern [52], future studies should use some strategies to improve data validity (e.g., consistency checks and completion time checks). Second, the PTSS is a self-report questionnaire, and social desirability may compromise answers. Third, the sample was comprised of mostly female participants, and future studies should encompass more heterogeneous samples. Due to the abovementioned limitations, the data cannot be generalized and should be analyzed cautiously.

Despite the study’s limitations, the findings revealed evidence of the PTSS’s reliability and construct validity among the Portuguese population, suggesting that the instrument can accurately measure the frequency of use of specific positive thinking skills.

## 5. Conclusions

This study has implications for practice. The PTSS is a brief measure that takes a few minutes to complete. This instrument can detect whether the population uses positive thinking skills and which of these are the most common in the general population or in specific samples.

As mentioned before, individuals usually assume that their thoughts are reflective of reality. They tend to have poorer mental health when they assume the negative version of reality, neglecting their potentialities and the optimistic view of a situation. For this reason, training one’s positive perspective of reality is crucial to amplifying positive thoughts and obtaining fewer negative conclusions. This work can balance an individual’s perspective on life events and improve their skills to deal with difficult situations (e.g., everyone makes mistakes). The positive approach can encourage one to use different ways to deal with situations than those usually employed, improving one’s mood or leading to better problem solving [23]. Developing cognitive reappraisal, which may involve replacing initially identified negative thoughts with positive thoughts, can be more adaptive and lead to better emotional regulation, adaptive behaviors, and problem solving [1]. Some studies have shown that positive thinking training reduces stress and anxiety [38]. Individuals who practice positive thinking can improve their physical and social functioning, increasing their emotional health and quality of life [39].

Therefore, using the appropriate instrument to measure positive thinking can improve the development of appropriate intervention programs for individual needs. The areas that scored lower on the scale can be improved through interventions to increase resilience levels. Studies show the effectiveness of the positive thinking training approach in enhancing resilience. Training in positive thinking leads to better thinking, increased resilience, and greater life satisfaction [53].

## Figures and Tables

**Table 1:** Description of the sample (*n* = 220).

	*n*	%
Education		
Higher Education (Bachelor’s, Master’s, Doctorate)	139	63.2
Secondary Education (10th to 12th year)	76	34.5
Elementary Education (7th to 9th year)	5	2.3
Sex		
Female	177	80.5
Male	43	19.5
Sexual Orientation		
Heterosexual	178	80.9
Homosexual	7	3.2
Bisexual	23	10.5
Pansexual	5	2.3
Undefined	7	3.2
Marital Status		
Single	188	85.5
Married	28	12.7
Divorced	4	1.8
Professional Situation		
Student	106	48.2
Employed	95	43.2
Unemployed	19	8.6

Note. *n* = Number of participants; % = percent of participants.

**Table 2:** Standardized regression weights for factor structure.

Items	*β* (Standardized)
1. Transform negative thoughts … *[Transformar pensamentos …]*	0.762
2. Highlight positive aspects … *[Destacar os aspetos positivos …]*	0.776
3. Interrupt pessimistic thoughts … *[Interromper pensamentos pessimistas …]*	0.687
4. Note the need to practice … *[Identificar … pensamento positivo.]*	0.735
5. Know how to break a problem … *[Saber como dividir um …]*	0.716
6. Initiate optimistic … *[Iniciar crenças otimistas … problema.]*	0.785
7. Natural ways … *[Criar formas … pessimistas.]*	0.820
8. Generate positive feelings … *[Gerar sentimentos positivos …]*	0.845

**Table 3:** Correlations between PTSS items.

	1.	2.	3.	4.	5.	6.	7.	8.
1. Item 1	-	0.66 **	0.45 **	0.53 **	0.46 **	0.49 **	0.50 **	0.57 **
2. Item 2		-	0.50 **	0.48 **	0.51 **	0.52 **	0.52 **	0.56 **
3. Item 3			-	0.49 **	0.36 **	0.44 **	0.47 **	0.55 **
4. Item 4				-	0.40 **	0.44 **	0.55 **	0.63 **
5. Item 5					-	0.61 **	0.55 **	0.52 **
6. Item 6						-	0.69 **	0.61 **
7. Item 7							-	0.70 **
8. Item 8								-

Note. ** p < 0.01; PTSS = Positive Thinking Skills Scales.

**Table 4:** Correlations between PTSS, PINTS, and RS-10.

	1.	2.	3.	4.	5.
1. PTSS	-	−0.19 **	0.52 **	0.43 **	0.53 **
2. PINTS		-	−0.28 **	−0.23 **	−0.28 **
3. RS-10 Total			-	0.93 **	0.93 **
5. Self-determination				-	0.74 **
6. Adaptability					-

Note. ** *p* < 0.01; PTSS = Positive Thinking Skills Scales; PINTS = Persistent and Intrusive Negative Thoughts Scale—Portuguese version; RS-10 = Resilience Scale-10—Portuguese version.

## Data Availability

The data presented in this study are available on request from the corresponding author. The data are not publicly available due to ethical, local reasons.

## References

[1] Johnson S.L. (2018). Therapist’s Guide to Clinical Intervention.

[2] Bekhet A., Zauszniewski J. (2013). Measuring use of Positive Thinking Skills Scale: Psychometric testing of a new scale. West. J. Nurs. Res..

[3] Noguchi K., Gohm C.L., Dalsky D.J. (2006). Cognitive tendencies of focusing on positive and negative information. J. Res. Pers..

[4] Dumitrache C.G., Windle G., Herrera R.R. (2015). Do social resources explain the relationship between optimism and life satisfaction in community-dwelling older people? Testing a multiple mediation model. J. Happiness Stud..

[5] McGrath P. (2004). The burden of ‘RA RA’ positive: Survivors’ and hospice patients’ reflection on maintaining a positive attitude to serious illness. Support Care Cancer.

[6] Naseem Z., Khalid R. (2010). Positive thinking in coping with stress and health outcomes: Literature review. J. Res. Reflect. Educ..

[7] Southwick S.M., Charney D.S. (2012). Resilience: The Science of Mastering life’s Greatest Challenges.

[8] Schimmel S. (2000). Vices, virtues and sources of human strength in historical perspective. J. Soc. Clin. Psychol..

[9] Bagherian S., Borhani F., Zadeh A.A., Ranjbar H., Solaimani F. (2012). The effect of distraction by bubble-making on the procedural anxiety of injection in Thalassemic school-age children in Kerman Thalasemia center. Adv. Nurs. Midwifery.

[10] Bohlmeijer E., Westerhof G. (2021). The model for sustainable mental health: Future directions for integrating positive psychology into mental health care. Front. Psychol..

[11] Fredrickson B.L., Tugade M.M., Waugh C.E., Larkin G.R. (2001). What good are positive emotions in crisis? A prospective study of resilience and emotions following the terrorist attacks on the United States on September 11th, 2001. J. Pers. Soc. Psychol..

[12] Seligman M.E.P., Csikszentmihalyi M. (2000). Positive psychology: An introduction. Am. Psychol..

[13] Lyubomirsky S., King L., Diener E. (2005). The Benefits of Frequent Positive Affect: Does Happiness Lead to Success?. Psychol. Bull..

[14] Cantor N., Norem J., Langston C., Zirkel S., Fleeson W., Cook-Flannagan C. (1991). Life tasks and daily life experience. J. Pers..

[15] Ong A., Bergeman C., Bisconti T., Wallace K. (2006). Psychological resilience, positive emotions, and successful adaptation to stress in later life. J. Pers. Soc. Psychol..

[16] Bunker S., Colquhoun D., Esler M., Hickie B.I., Hunt D., Jelinek V.M., Oldenburg B.F., Peach H.G., Ruth D., Tennant C.C. (2003). “Stress” and coronary heart disease: Psychosocial risk factors. Med. Stud. J. Aust..

[17] Pedersen A.F., Bovbjerg D.H., Zachariae R., Contrada R.J., Baum A. (2011). Stress and susceptibility to infectious disease. The Handbook of Stress Science: Biology, Psychology, and Health.

[18] Stojanovich L., Marisavljevich D. (2008). Stress as a trigger of autoimmune disease. Autoimmun. Rev..

[19] Tugade M.M., Frederickson B.L. (2004). Resilient persons use positive emotions to bounce back from negative emotions experiences. J. Pers. Soc. Psychol..

[20] Achat H., Kawachi I., Spiro A., DeMolles D.A., Sparrow D. (2000). Optimism and depression as predictors of physical and mental health functioning: The Normative Aging Study. Ann. Behav. Med..

[21] Shokrpour N., Sheidaie S., Amirkhani M., Bazrafkan L., Modreki A. (2021). Effect of positive thinking training on stress, anxiety, depression, and quality of life among hemodialysis patients: A randomized controlled clinical trial. J. Educ. Health Promot..

[22] Craske M.G. (2017). Cognitive-Behavioral Therapy.

[23] Moore R.G., Garland A. (2003). Cognitive Therapy for Chronic and Persistent Depression.

[24] Masten A.S. (2001). Ordinary magic: Resilience process in development. Am. Psychol..

[25] Masten A.S., Read M.J., Snyder C.R., Lopez S.J. (2002). Resilience in development. Handbook of Positive Psychology.

[26] Steinhardt M., Dolbier C. (2008). Evaluation of a resilience intervention to enhance coping strategies and protective factors and decrease symptomatology. J. Am. Coll. Health.

[27] Matel-Anderson D.M., Bekhet A.K. (2019). Psychometric properties of the positive thinking skills scale among college students. Arch. Psychiatr. Nurs..

[28] Yates T.M., Tyrell F., Masten A.S. (2015). Resilience theory and the practice of positive psychology from individuals to societies. Positive Psychology in Practice: Promoting Human Flourishing in Work, Health, Education, and Everyday Life.

[29] Bekhet A.K., Garnier-Villarreal M. (2017). The positive thinking skills scale: A screening measure for early identification of depressive thoughts. Appl. Nurs. Res..

[30] Wong S. (2012). Negative thinking versus positive thinking in a Singaporean student sample: Relationships with psychological well-being and psychological maladjustment. Learn. Individ. Differ..

[31] Ehring T., Zetche U., Weidacker K., Schonfeld S., Ehlers A. (2011). The Perseverative Thinking Questionnaire (PTQ): Validation of a content-independent measure of repetitive negative thinking. J. Behav. Ther. Exp. Psychiatry.

[32] Hur J., Heller W., Kern J.L., Berenbaum H. (2017). A bi-factor approach to modeling the structure of worry and rumination. J. Exp. Psychopathol..

[33] McEvoy P.M., Brans S. (2013). Common versus unique variance across measures of worry and rumination: Predictive utility and mediational models for anxiety and depression. Cogn. Ther. Res..

[34] Gonzalez-Robles A., Díaz-García A., Miguel C., García-Palacios A., Botella C. (2018). Comorbidity and diagnosis distribution in transdiagnostic treatments for emotional disorders: A systematic review of randomized controlled trials. PLoS ONE.

[35] Akin A., Uysal R., Akin U. (2015). The validity and reliability of the Turkish version of the Positive Thinking Skills Scale. Eur. J. Educ..

[36] Bekhet A.K., Nakhla V., Gohar I.E., Oudeh R., Gergis M., Malik N. (2020). Cross-Cultural adaptation and psychometric properties of the Arabic version of the Positive Thinking Skills Scale. Issues Ment. Health Nurs..

[37] Bekhet A.K., Matel-Anderson D. (2017). Risk and protective factors in the lives of caregivers of persons with autism: Caregivers’ perspectives. Perspect. Psychiatr. Care.

[38] Barjoee L.K., Amini N., Keykhosrovani M., Shafiabadi A. (2022). Effectiveness of positive thinking training on perceived stress, metacognitive beliefs, and death anxiety in women with breast cancer: Perceived stress in women with breast cancer. Arch. Breast Cancer.

[39] Sadeghloo A., Shamsaee P., Hesari E., Akhondzadeh G., Hojjati H. (2019). The effect of positive thinking training on the quality of life of parents of adolescent with thalassemia. Int. J. Adolesc. Med. Health.

[40] Magson N.R., Rapee R.M., Fardouly J., Forbes M.K., Richardson C.E., Johnco C.J., Oar E.L. (2019). Measuring repetitive negative thinking: Development and validation of the Persistent and Intrusive Negative Thoughts Scale (PINTS). Psychol. Assess..

[41] Peixoto M., Cunha O. (2021). Translation and validation for the portuguese adult population of the Persistent and Intrusive Negative Thoughts Scale: Assessing measurement invariance. Int. J. Cogn. Ther..

[42] Jardim J., Pereira A., Bártolo A. (2021). development and psychometric properties of a scale to measure resilience among Portuguese university students: Resilience Scale-10. Educ. Sci..

[43] World Medical Association (2013). World Medical Association Declaration of Helsinki: Ethical principles for medical research involving human subjects. JAMA.

[44] Marôco J. (2014). Análise de Equações Estruturais: Fundamentos Teóricos, Software & Aplicações.

[45] Netemeyer R.G., Bearden W.O., Sharma S. (2003). Scaling Procedures: Issues and Applications.

[46] Field A. (2017). Discovering Statistics Using IBM SPSS Statistics.

[47] Gravetter F., Wallnau L. (2014). Essentials of Statistics for the Behavioral Sciences.

[48] Yue Z., Liang H., Qin X., Ge Y., Xiang N., Liu E. (2022). Optimism and survival: Health behaviors as a mediator-a ten-year follow-up study of Chinese elderly people. BMC Public Health.

[49] Seligman M.E.P., Rashid T.A.C., Parks A.C. (2006). Positive psychotherapy. Am. Psychol..

[50] Sabouri F., Rambod M., Khademian Z. (2023). The effect of positive thinking training on hope and adherence to treatment in hemodialysis patients: A randomized controlled trial. BMC Psychol..

[51] Akbarinejhad H.S., Faroughi P. (2021). Comparison of the effectiveness of positive thinking training and acceptance and commitment therapy on quality of life and resilience of people living with HIV. HIV AIDS Rev..

[52] Paas L.J., Morren M. (2018). Please do not answer if you are reading this: Respondent attention in online panels. Mark. Lett..

[53] Taherkhani Z., Kaveh M.H., Mani A., Ghahremani L., Khademi K. (2023). The effect of positive thinking on resilience and life satisfaction of older adults: A randomized controlled trial. Sci. Rep..

